# Youth with severe obesity do not demonstrate increased eating disorder symptoms following family-based behavioral obesity treatment

**DOI:** 10.1007/s40519-026-01839-3

**Published:** 2026-03-14

**Authors:** Yngvild S. Danielsen, Hanna F. Skjåkødegård, Denise Wilfley, Rachel P. K. Conlon, Helge Molde, Vilde Aabel Skodvin, Petur B. Juliusson

**Affiliations:** 1 Department of Clinical Psychology, University of Bergen, Bergen, Vestland Norway; 2 Regional Department of Eating Disorders, Haukeland University Hospital, Bergen, Vestland Norway; 3 Children and Youth Clinic, Haukeland University Hospital, Bergen, Vestland Norway; 4 Department of Psychiatry, Washington University, St. Louis, Missouri USA; 5 School of Medicine, University of Pittsburgh, Pittsburgh, Pennsylvania USA; 6 Department of Clinical Science, University of Bergen, Bergen, Vestland Norway; 7 Counseling on Eating Disorders (ROS), Bergen, Vestland Norway; 8 Department of Health Registry Research and Development, Norwegian Institute of Public Health, Bergen, Vestland Norway

**Keywords:** Eating disorder, Youth, Obesity, Family-based behavioral treatment

## Abstract

**Purpose:**

To investigate eating disorder symptoms before and after family-based behavioral treatment for severe obesity in youth.

**Methods:**

Eating disorder symptoms were measured pre- and posttreatment in 74 youth (age: 10–18 years; 58% female; mean body mass index standard deviation score [BMI SDS]: 3.11) enrolled in a 17–session treatment for severe obesity. Symptoms were assessed using the Youth-Eating Disorder Examination Questionnaire (Y-EDE-Q), including a global score and sub-scales for dietary restraint, eating-, weight- and shape concerns. Robust linear mixed models and generalized mixed models were utilized to measure change over time.

**Results:**

There was no significant change in global eating disorder score over time (mean (SD) pre 1.82(1.07) and post 1.67(1.08); beta = − 0.16, p = 0.69). Before treatment, 20.3% of the youth scored above the clinical cut-off for eating disorder risk (> 2.5) on the Y-EDE-Q, compared to 13.5% after treatment (beta = − 0.81, p = 0.25). However, no one received a clinical eating disorder diagnosis at any time point. Before treatment, 4 youth reported ≥ 1 objective binge episode per week, compared to no one post-treatment. A significant reduction in shape concern was observed (beta = − 0.29, p = 0.036). No changes were found for dietary restraint, eating- or weight concerns. Global eating disorder- and dietary restraint scores pre-treatment did not predict changes in BMI SDS from pre- to post-treatment.

**Conclusion:**

There was no evidence of an increase in eating disorder symptoms in the sample overall during family-based behavioral treatment for severe obesity, and improvements were seen for shape concerns.

**Level of evidence:**

Level IV: Evidence obtained from multiple time series with intervention.

**Supplementary Information:**

The online version contains supplementary material available at 10.1007/s40519-026-01839-3.

## Introduction

Family-based behavioral treatment (FBBT) is recommended as the first line of treatment for obesity in children and youth [1]. However, concerns have been raised that behavioral weight management for obesity, including goals related to dietary quality and caloric ranges, may increase eating disorder symptoms in youth and pose a risk for developing an eating disorder [2–4].

Youth with obesity have an elevated risk of developing eating disorders, as obesity and eating disorders share several risk factors [5–10]. A study of adolescents seeking treatment for restrictive eating disorders found that 36.7% had a previous history of weight above the 85th percentile [10], indicating that higher weight quite often could be involved in the etiology of restrictive eating disorders. Treatment seeking youth with severe obesity also often exhibit elevated levels of binge eating behaviors and different sub-threshold eating disorder symptoms, as well as body image concerns [11–15]. Further, some might have a full-blown eating disorder in need of specialized eating disorder treatment [9, 11]. Therefore, screening for and monitoring eating disorder symptoms in obesity care is crucial, especially during adolescence, a period of heightened risk for eating disorders regardless of weight status [16, 17]. Eating disorder screening is due to this recommended in guidelines for evaluation and treatment of adolescent obesity to ensure tailored care [1]. In addition, it is imperative to ensure that obesity treatments do not trigger or worsen eating disorder symptoms among youth, and to monitor these symptoms closely during treatment.

A 2019 meta-analysis examining various lifestyle treatments for childhood and adolescent obesity identified 13 studies examining eating disorder symptoms before and after treatment [18]. One study found an increased risk of eating disorders following lifestyle treatment, while three studies reported a reduced risk, and 6 studies showed no change in symptoms [18]. Further, 5 studies reported on the prevalence of binge eating episodes before and after treatment, all finding a general reduction of episodes. However, one study found that four out of 47 participants developed binge eating episodes during treatment [19]. Recently published data from a behavioral obesity intervention for adolescents employing a low energy dietary approach likewise found a general decrease in eating disorder symptoms at 52-weeks follow-up [20]. However, 9% of the participants had an increase in symptoms from below to above clinical cut-off for eating disorders from start of treatment to the 52-week follow-up [20]. Summarized, these studies indicate that while eating disorder symptoms generally decrease with lifestyle treatments for obesity, incidents of eating disorder development or persistence of symptoms still occur. Moreover, most studies reporting on changes in eating disorder risk following obesity treatment have relatively short follow-up periods and therefore cannot conclude about long-term risk [21].

FBBT for obesity is more intensive and psychologically oriented than standard lifestyle interventions and aims to reduce eating disorder symptoms by establishing regular eating patterns, family meals, planning ahead, creating less triggering environments for eating in absence of hunger, promoting support from parents and social networks, addressing body image concerns, and practicing emotion regulation and interpersonal skills [14, 22].

Nevertheless, FBBT for severe obesity in youth often aims to achieve weight reduction and focuses on dietary quality and energy restriction. Monitoring weight and eating habits are important treatment tools [22]. These aspects of the treatment could potentially increase dietary restraint, defined as a cognitive, willful effort to restrict energy intake or avoid certain types of food [23].

In general, studies examining the relationship between dietary restraint and binge eating within the context of behavioral obesity treatment do not indicate that increased dietary restraint causes more binge eating or other eating disorder symptoms [23]. Few studies, however, have been conducted in youth [18, 23, 24]. A review of all types of pediatric lifestyle obesity interventions found that dietary restraint most often increased following treatment (10 studies at post intervention, 6 studies at follow-up). The remaining studies reported no changes in dietary restraint (7 post intervention and 5 at follow-up). All the studies presenting findings concerning other eating disorder symptoms (e.g. binge eating) or eating disorder risk factors (e.g. body image concerns or depression) found improvements or no change [24]. However, one German study of 111 youth with overweight attending lifestyle treatment found that higher baseline levels of dietary restraint predicted lower weight reduction after 12 months treatment [25]. Overall, these findings indicate that dietary restraint in the context of specialized lifestyle/behavioral obesity treatment generally does not increase eating disorder risk, at least not in the short term. Further, it highlights some challenges with the construct of dietary restraint and how it is measured within obesity care, as it could represent both adaptive and non-adaptive thoughts and behaviors [23]. Only three of the studies in the above mentioned review examined FBBT interventions, and all of these included younger children (ages 7–13 years) [24].

As summarized above, only a few studies have examined eating disorder symptoms, and risk in FBBT for youth with obesity. To address this knowledge gap, the current study examines pre-post changes in eating disorder symptoms in youths attending a 17–session FBBT for severe obesity. Further, it explores whether the global level of eating disorder symptoms and dietary restraint before starting treatment influence pre-post changes in Body Mass Index Standard  Deviation Scores (BMI SDS). Dietary restraint is hypothesized to increase from pre- to post treatment, while the global eating disorder symptoms and eating-, weight- and shape concerns are hypothesized to remain stable or decrease. Further, a reduction in the number of youths reporting weekly objective binge eating episodes is expected.

## Methods

### Design

This paper includes secondary analyses of data from the Family-Based behavioral Obesity treatment trial (The FABO-study), comparing the effectiveness of FBBT to treatment as usual (TAU) for children and adolescents with severe obesity [22, 26].

**Fig. 1:**
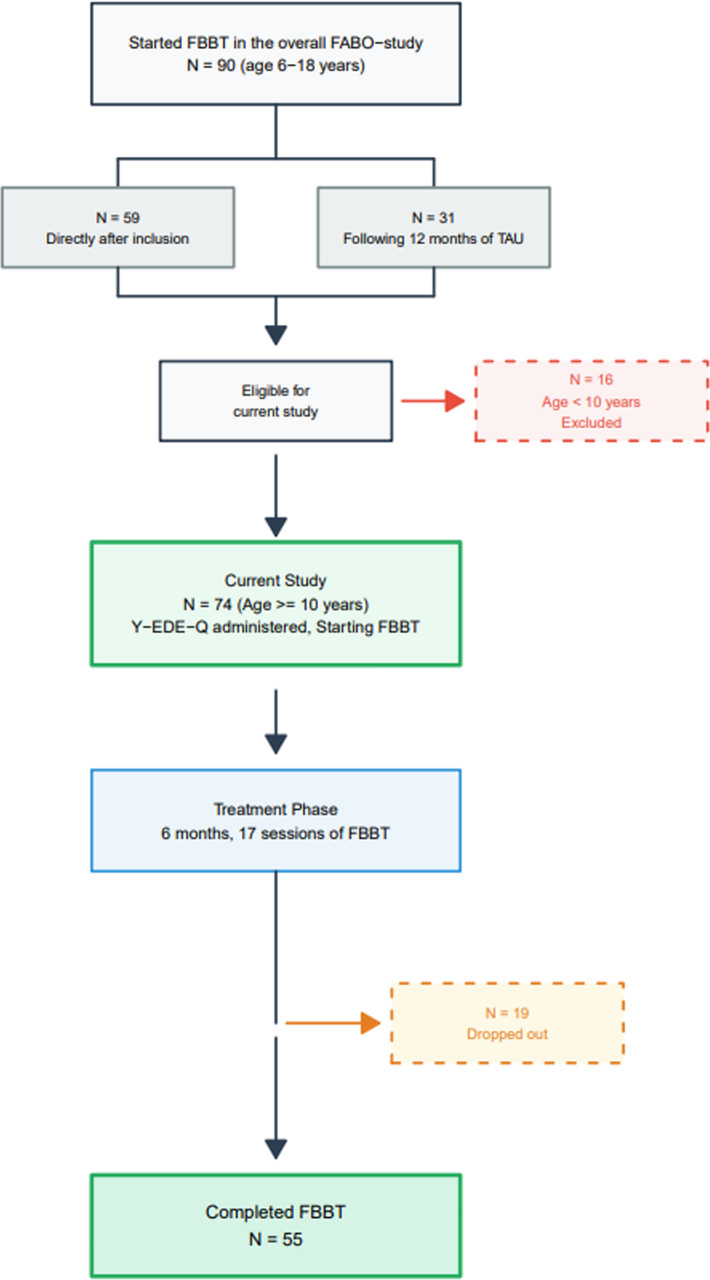
Flowchart of the study

The Y-EDE-Q was only administrated to children above 10 years of age in the FABO-study, resulting in 74 adolescents being included in the present study. The questionnaire data analyzed in the present study was collected before the first FBBT session (pre-treatment) and after the last of the 17 sessions (post-treatment). Some of the adolescents had received TAU before entering the FBBT (N = 31; Arm B in FABO), according to the wait-list design for FBBT of the FABO-study (see Fig. 1). The adolescents in the study were enrolled between 2014 and 2018. The FABO-trial, including pre-calculations of statistical power for the randomized controlled trial (RCT), is described in more detail in the protocol paper by Skjåkødegård et al. [26].

### Participants

All participants were referred to a tertiary obesity outpatient clinic in Bergen, Norway, by their general practitioner. International Obesity Task Force (IOTF) cut-offs for severe obesity in youth were used as inclusion criteria for the study (or obesity with co-morbidity) [27]. Exclusion criteria included severe somatic or psychiatric illness influencing weight (including eating disorders), inability to attend weekly sessions, or participation in other obesity treatments. If participants scored above cut-off on the Y-EDE-Q, or reported purging behaviors, their treatment provider was instructed to follow up by conducting a clinical interview about eating disorder symptoms based on the ICD-10 or DSM-5 criteria. If further diagnostics and follow-up on eating pathology were indicated, the youth should be referred for assessment by the child and adolescent psychiatric team at the hospital. Applying these procedures none of the participants were diagnosed with an eating disorder. Of the 74 adolescents starting FBBT treatment, 19 ended prematurely. In total the dataset had 25.7% missing observations on the Y-EDE-Q.

### Treatment

The 17-session FBBT delivered in this study was based on the work of Epstein and Wilfley [28, 29]. The treatment aims to establish healthy lifestyle behaviors and attitudes within the family system to support weight management. Key target areas include eating habits, physical activity, screen use, and sleep. Parents are involved both in supporting their child and by targeting their own lifestyle behaviors and communication about body and weight within the family. FBBT uses behavioral and cognitive techniques to promote self-regulation, such as goalsetting, self-monitoring, planning, systematic rewards, problem solving, challenging negative automatic thoughts, practicing self-assertion skills and expressing needs. It supports parents in creating functional family rules and providing practical and emotional support for their children. The treatment included the Traffic light eating and activity plan, providing information about the nutritional value and energy density of different foods, and energy expenditure in different activities [30, 31]. For adolescents with severe obesity, the goal was a weight reduction of approximately 1 kg/month during the treatment period. At all sessions, weight was monitored for adolescents and parents, along with self-monitoring of eating habits and other target behaviors between sessions. Weight graphs were used to monitor and discuss changes over time. Regularity of meals formed the basis for changes in eating behaviors. The 17 sessions were delivered weekly in 45–60 min meetings with the same clinician. As a main principle the youth and his/her parent(s) attended the sessions together. The clinicians had professional backgrounds across disciplines (nurses, pediatricians, psychologists, physiotherapists and nutritionists) and attended training workshops by collaborating psychologists from Denise Wilfley’s lab at Washington University in St. Louis and participated in monthly supervision meetings.

### Measures and outcome

Demographic information was collected using a standardized questionnaire, while weight and height were measured at the clinic before and after the treatment by trained supervisors for calculation of Body Mass Index (BMI; kg/m^2^). Norwegian growth references were used for calculating BMI standard deviation scores (SDS) [32].

#### Eating disorder symptoms

*The Youth Eating Disorder Examination-Questionnaire (Y-EDE-Q)* is the primary outcome measure in the current study [33]. It is a 39-item self-report measure of eating disorder symptoms over the last 28 days. A total of 26 statements about eating patterns and attitudes and feelings about body and weight are rated on a 7–point Likert scale. Additionally, 13 questions about the prevalence of specific eating disorder symptoms are included [34, 35]. The Y-EDE-Q has been validated against the ChEDE for the assessment of eating disorder symptoms in adolescents with overweight/obesity [35]. Scoring includes a global score and subscales for dietary restraint, eating-, weight- and shape concerns, as well as questions about the number of objectively large eating episodes with and without loss of control during the last 28 days. A clinical cut-off of 2.5 on the Y-EDE-Q was used to indicate risk of eating disorders [36]. Cronbach’s alpha for the Y-EDE-Q global score in our sample was 0.93. For the sub-scales Cronbach’s alphas were 0.62 for Dietary restraint, 0.70 for Eating concerns, 0.77 for Weight concerns and 0.90 for Shape concerns.

### Data analysis

To estimate changes over time, robust linear mixed models were utilized. All models were random intercept, fixed slope models in which measurement occasions (time) is nested within subjects. Random intercepts allow each subject their own baseline score, while the fixed slope parameter estimates the average change over time across subjects. Linear mixed models are flexible, allowing for missing data across subjects and/or time [37]. For the main analyses of the linear mixed models, we applied the “robustlmm” package, and for binary outcomes, we applied the “lme4” package in R (R Foundation for Statistical Computing, 2023, version 4.3.1). The linear multilevel models have assumptions of normality of residuals and homoscedasticity; thus, evaluating the residual distributions from our model’s fit, we applied the plots from the “robustlmm” package and the “DHARMa” package for the binary outcomes. The plots are available in Supplementary appendix A.

## Results

Table 1 provides the means, standard deviations, and prevalence percentages for the variables included in the study Tables 2 and 3 present the results. Before treatment, 20.3% (N = 15) of the youth scored above the clinical cut-off for risk of eating disorders (> 2.5) on the Y-EDE-Q, compared to 13.5% (N = 10) after treatment. However, when we fitted a multilevel binomial regression model, with eating disorders (> 2.5) as the dependent variable, the fixed effect of time was not significant (beta = − 0.81, p = 0.25). In addition, neither sex (beta = 1.75, p = 0.10) nor age (beta = 0.26, p = 0.24) was significant as predictors in the model.

**Table 1:** Characteristics of the participants

N = 74	T1	T2
Age (mean ± SD, min–max)	14.2 ± 2.3 9.9–18.6	
Sex boys/girls (%girls)	31/43 (58%)	
BMI (mean ± SD) BMI SDS (mean ± SD)	34.0 ± 4.3 3.11 ± 0.5	34.5 ± 9.4 3.07 ± 0.7
Global Y-EDE-Q score (mean ± SD)	1.8 ± 1.1	1.7 ± 1.0
Y-EDE-Q dietary restraint (mean ± SD)	1.5 ± 1.0	1.7 ± 1.2
Y-EDE-Q eating concern (mean ± SD) Y-EDE-Q weight concern (mean ± SD)	1.1 ± 1.0 2.3 ± 1.3	0.9 ± 0.9 2.0 ± 1.3
Y-EDE-Q shape concern (mean ± SD)	2.3 ± 1.6	2.0 ± 1.5
Y-EDE-Q global score > 2.5 (N, %)	15 (20.3%)	10 (13.5%)
Y-EDE-Q global score > 4.0 (N, %)	0 (0%)	1 (1.3%)
Y-EDE-Q weekly objective binge eating episodes (N, %)	4 (5.4%)	0 (0%)
Y-EDE-Q purging last 28 days (N, %)	2 (2.7%)	3 (4.0%)

BMI SDS, body mass index z-scores; BMI, body mass index; SD, standard deviation; Scores on the Y-EDE-Q subscales can range from 0 to 6

**Table 2:** Linear mixed model of changes in the Y-EDE-Q sub-scales over time (pre- to post-treatment)

N = 60	Dietary restraint			Eating concern			Shape concern			Weight concern		
Predictors	Estimates	CI	p	Estimates	CI	p	Estimates	CI	p	Estimates	CI	p
(Intercept)	0.27	− 1.40 to 1.93	0.755	− 0.21	− 1.63 to 1.21	0.774	− 0.85	− 3.32 to 1.62	0.500	− 0.48	− 2.53 to 1.56	0.642
Time	0.18	− 0.06 to 0.42	0.138	− 0.18	− 0.40 to 0.04	0.112	− 0.29	− 0.56 to − 0.02	0.036	− 0.24	− 0.55 to 0.06	0.121
Sex [female]	0.15	− 0.36 to 0.66	0.567	0.43	0.00–0.86	0.048	0.75	− 0.00 to 1.50	0.050	0.50	− 0.11 to 1.12	0.109
Age T1	0.06	− 0.05 to 0.17	0.259	0.08	− 0.02 to 0.17	0.104	0.20	0.04–0.37	0.017	0.19	0.05–0.32	0.007
Random effects												
σ^2^	0.33			0.28			0.40			0.55		
τ_00_	0.71_id1_			0.48_id1_			1.74_id1_			1.00_id1_		
ICC	0.68			0.63			0.81			0.65		
N	60_id1_			60_id1_			60_id1_			60_id1_		
Observations	105			103			103			104		
Marginal R^2^/Conditional R^2^	0.033/0.694			0.101/0.668			0.148/0.842			0.141/0.695

**Table 3:** Linear mixed model of changes in the Y-EDE-Q global score and BMI SDS from pre- to post-treatment

N = 59	Global score > 2.5			Global score			T 1 and T 2 BMI SDS		
Predictors	Odds ratios	CI	p	Estimates	CI	p	Estimates	CI	p
(Intercept)	0.00	0.00–6.14	0.133	−0.33	−2.00 to 1.33	0.693	1.32	0.73–1.91	< 0.001
Time	0.44	0.11–1.75	0.245	−0.16	−0.37 to 0.04	0.125	−0.07	−0.13 to −0.00	0.038
Sex [female]	5.76	0.72–46.27	0.100	0.48	−0.03 to 0.99	0.062	0.52	0.34–0.70	< 0.001
Age T1	1.31	0.84–2.05	0.236	0.14	0.02–0.25	0.017	0.11	0.07–0.15	< 0.001
Y-EDE-Q global score							0.04	−0.04 to 0.12	0.279
Y-EDE-Q dietary restraint							−0.02	−0.09 to 0.04	0.471
Random effects									
σ^2^	3.29			0.23			0.02		
τ_00_	5.16_id1_			0.75_id1_			0.10_id1_		
ICC	0.61			0.77			0.83		
N	59_id1_			59_id1_			59_id1_		
Observations	101			101			101		
Marginal R^2^/Conditional R^2^	0.133/0.662			0.142/0.800			0.556/0.923		

BMI SDS = Body mass index standard deviation scores based on the Bergen Growth Study (28); The Scores on the Y-EDE-Q subscales can range from 0-6; T1 = pre-treatment, T2 = post treatment

Before treatment, four youths reported an average of more than one weekly binge eating episode (i.e., consuming an objectively large amount of food and experiencing a sense of loss of control) during the past month, compared to none at the end of treatment. None of the participants received a clinical eating disorder diagnosis at any time point. Supplementary appendix B visualizes the Y-EDE-Q item distributions by subscale before and after treatment.

For the global Y-EDE-Q score, the total explanatory power was high (conditional R^2^ = 0.80). The marginal R^2^ was low (0.14), and the ICC was 0.77. The fixed effects of time and sex was not significant (beta = − 0.16, p = 0.69; beta = 0.48, p = 0.13). The fixed effect of age on the other hand was significant (beta = 0.14, p = 0.017), indicating that older participants had more global eating disorder concerns, independent of time.

For the sub-scale “Dietary restraint”, the total explanatory power was reasonably high (conditional R^2^ = 0.69). The marginal R^2^ was low (0.03), and the ICC was 0.68. Neither the fixed effect of time (beta = 0.18, p = 0.14) nor sex was significant (beta = 0.15, p = 0.56), and neither was the fixed effect of age (beta = 0.06, p = 0.26).

For the sub-scale “Eating concern”, total explanatory power was reasonably high (conditional R^2^ = 0.67). The marginal R^2^ was low (0.10), and the ICC was 0.63. The fixed effect of time was not significant (beta = − 0.18, p = 0.11). The fixed effect of sex was significant (beta = 0.43, p = 0.048), indicating that girls had relatively more eating concern than boys, independent of time. The fixed effect of age was not significant (beta = 0.08, p = 0.10).

For the sub-scale “Shape concern”, the total explanatory power was high (conditional R^2^ = 0.84). The marginal R^2^ was low (0.15), and the ICC was 0.81. The fixed effect of time was significant (beta = − 0.29, p = 0.036), showing that shape concerns decreased over time. The fixed effect of sex was significant (beta = 0.75, p = 0.05), indicating that girls had relatively more shape concerns than boys independent of time. The fixed effect of age was also significant (beta = 0.20, p = 0.017), indicating that older participants had more shape concerns, independent of time.

For the measure “Weight concern”, the total explanatory power was reasonably high (conditional R^2^ = 0.70). The marginal R^2^ was low (0.14), and the ICC was 0.65. The fixed effect of time was not significant (beta = − 0.48, p = 0.64). The fixed effect of sex was not significant (beta = − 0.24, p = 0.12). The fixed effect of age was significant (beta = 0.19, p = 0.007), indicating that older participants had more weight concerns, independent of time.

For the measure “BMI SDS”, the total explanatory power was high (conditional R^2^ = 0.92). The marginal R^2^ was reasonable high (0.56), and the ICC was 0.83. The fixed effect of time was significant (beta = − 0.07, p < 0.001). The fixed effect of sex was significant (beta = − 0.52, p < 0.001), indicating that girls had relatively higher BMI SDS than boys, independent of time. The fixed effect of age was significant (beta = 0.11, p < 0.001), indicating that older participants had higher BMI SDS, independent of time. Both “Dietary restraint” and Global Y-EDE-Q were non-significant predictors for change in BMI SDS in treatment.

## Discussion

The current study investigates self-reported eating disorder symptoms in 74 youths aged 10–18 years before and after family-based behavioral treatment for severe obesity. Our results suggest that at a group level there was no significant change in global eating disorder symptoms nor dietary restraint scores on the Y-EDE-Q from before and after FBBT for severe obesity. Further, the percentage of adolescents scoring above the clinical cut-off for eating disorder risk was not significantly different before and after treatment. However, there was an overall improvement in shape concerns, and none reported weekly binge eating episodes at the end of treatment. The level of eating disorder symptoms before treatment did not predict changes in BMI SDS.

### Eating disorder risk during FBBT treatment

Screening for eating disorders and monitoring eating disorder symptoms is described as essential in guidelines for obesity care for children and adolescents [1]. Thus, acknowledging the need for targeted eating disorder treatment for co-morbid eating disorders and obesity. The present study in line with this assessed eating disorders at baseline and had diagnosed eating disorders as exclusion criterion for participation. In this context the findings do not indicate a general elevated risk for the development of eating disorders in youths enrolled in a FBBT intervention for obesity. This aligns with most previous studies examining eating disorder risk in lifestyle treatment for children and adolescents with obesity [18, 24]. It is important, however, to keep in mind that results from the present study cannot be generalized to young people enrolling in treatment for obesity with a co-morbid eating disorder.

### Dietary restraint and binge eating episodes

While some previous studies have reported increased scores on measures of dietary restraint, this was not the case in the present study [23, 24]. However, 2 single items on the Y-EDE-Q had a mean increase from pre- to post treatment: Item 4 “On how many of the past 28 days have you tried to stick to strict rules about your eating in order to change your shape or weight; for example, only letting yourself eat a certain type or amount of food, or certain number of calories?”, and item 5 asking about whether you try to avoid certain types of food in order regulate body shape or weight. This finding indicates that there was a general tendency to use more cognitive efforts to control eating at the end of treatment than at the start. A central question here is whether higher scores on these two items in the context of FBBT constitutes a general risk factor for development of other eating disorder symptoms and a too high degree of inflexibility and rigidity, or does it represent a shift from stimulus- and affect-driven behavior to more cognitive, goal-directed behavior as promoted in behavioral treatment for obesity. In the present study all other items on the Y-EDE-Q demonstrated a mean improvement or remained stable at the end of treatment, lending no support for a general deterioration in eating disorder symptoms. It is however important to keep in mind that we do not know whether scores and symptoms would be different at a later time-point after ended treatment.

The focus on regularity of meals might be a protective factor against binge eating in the FBBT treatment. No findings from the current study supported that regular meals with moderate energy restriction triggered binge eating episodes. However, measures were only obtained before and after treatment, and information is lacking about fluctuations in energy intake and eating behaviors during the treatment. The attainment of the weight goals also varied among the youths participating, with only 20% having a BMI SDS reduction above − 0.25 often considered as a clinically significant weight reduction [38]. In addition, participants with binge eating disorder were not included in the study and might have been more at risk.

### Tailoring treatment based on eating disorder symptoms

At the start of treatment 20.3% reported eating disorder symptoms above the Norwegian recommended general eating disorder risk cut-off on the EDE-Q, and the percentage did not significantly decrease during treatment [36]. This indicates that several of the youths still had eating disorder symptoms at the end of treatment, even though they did not have a full-blown eating disorder. Providing eating disorder-focused treatments for these children with heightened symptoms, as well as addressing sub-clinical eating disorder presentations, might be an important adjustment of their health care. This could include addressing psychological risk factors for eating disorders by working more in-depth therapeutically with emotions, relationships, self-evaluations and body image [14, 39]. The findings also highlight the need for systematic assessment of eating disorders in obesity care to identify individuals at risk for eating disorder development.

### Challenges with eating disorder assessment in the context of obesity care

While several studies have examined EDE-Q and Y-EDE-Q scores in different populations, there does not seem to be an established consensus on a clinical cut-off score indicating the likely presence of an eating disorder among adolescents with obesity [36, 40]. Samples of participants with obesity tend to score higher than other clinical eating disorder populations on the EDE-Q [36, 41]. This could be due to a higher prevalence and severity of eating disorders but might also be due to a different conceptualization of dietary restraint within obesity treatment compared to other eating disorder treatments. While cognitive control of eating might be seen as preferable within behavioral obesity treatment, overvaluation of this type of control is also seen as central to eating disorder pathology. In addition, some questions about weight and shape concerns might refer to different thoughts and feelings depending on actual weight status such as “on how many of the last 28 days have you been feeling fat?” (From Y-EDE-Q). The single Y-EDE-Q items with the highest scores in the present study and thus contributing most to the Global eating disorder score were: Desire to lose weight, dissatisfaction with weight and shape and discomfort when self- or others see your body. Scores on these items on a group level improved or remained stable at the end of treatment.

The prevalence/frequency of loss of control eating is found to be higher when measured by the Y-EDE-Q self-report questionnaire compared to the Child Eating Disorder Examination interview [40]. This could be due to difficulties for children and adolescents in conceptualizing both loss of control and what is considered an objectively large amount of food. The Y-EDE-Q used in the present study included a visualization of portions and written explanations of these phenomena to aid youth’s understanding. Still, four participants prior to treatment reported more than one weekly binge eating episode with loss of control during the last month on the Y-EDE-Q, but none of these received a clinical BED diagnosis when following the assessment routines at the clinic. This could indicate that the Y-EDE-Q has the sensitivity to detect objective binge eating episodes among adolescents, but that specificity for identifying BED might be poorer.

## Strengths and limits

Due to the limited sample size above 10 years and reporting on Y-EDE-Q in the FABO-study we could not analyze Y-EDE-Q data in a randomized design in this paper. The current study therefore has no control group that did not receive the intervention, and the findings must be interpreted with caution. Further, the drop-out rate was quite high resulting in missing data about eating disorder symptoms for a sub-group. Some of these participants might potentially have had an increase in eating disorder symptoms. Larger randomized controlled trials or aggregated datasets from several trials are needed to further explore the risk of eating disorders in the context of obesity treatments and identify factors associated with higher risk. Further, knowledge about which specific treatment components might exacerbate eating disorder risk is needed, as well as studies with longer follow-up periods.

The lack of standardized interviews for eating disorder diagnostics at the outpatient clinic could mean that there were inaccuracies in the diagnosis of eating disorders and constitutes a weakness of the study. Another weakness of the study is that the dataset did not include information about medications that might influence weight- or eating, however, no medications for weight reduction were prescribed to any participant during the study.

## What is already known about the subject?

Children enrolled in lifestyle interventions for obesity do not on a group level demonstrate increased eating disorders symptoms at the end of treatments, even though some incidents of eating disorder development might occur, as well as increased levels of dietary restraint.

## What this study adds

Youth with severe obesity and without a pre-existing eating disorder did not on a group level increase eating disorder symptoms during FBBT. However, a substantial proportion of individuals presenting for FBBT scored above the clinical cut-off for eating disorder risk on the Y-EDE-Q both before and after the intervention. Systematic assessment of eating disorder symptoms in obesity care is important for identifying those who need eating disorder treatment and for modifying treatments for those with sub-clinical eating disorder symptoms. There is also a need for developing more targeted tools for the assessment of eating disorder symptoms and eating pathology in children and adolescents with obesity.

## Supplementary Information

Below is the link to the electronic supplementary material.

Supplementary Material 1.

Supplementary Material 2.

## Data Availability

The raw/processed data required to reproduce the above findings cannot be shared at this time due to legal/ethical reasons but could be made available upon request for control purposes until the ethical approval requires them deleted.
