# Supplementary material for: Youth with severe obesity do not demonstrate increased eating disorder symptoms following family-based behavioral obesity treatment
Source: Eat Weight Disord. 2026 Mar 14;31(1):38. doi: 10.1007/s40519-026-01839-3 (PMC13099665; doi:10.1007/s40519-026-01839-3)

**Appendix A: plots of model residuals**

1. **Global score > 2.5** model residuals using the “DHARMa” package in R


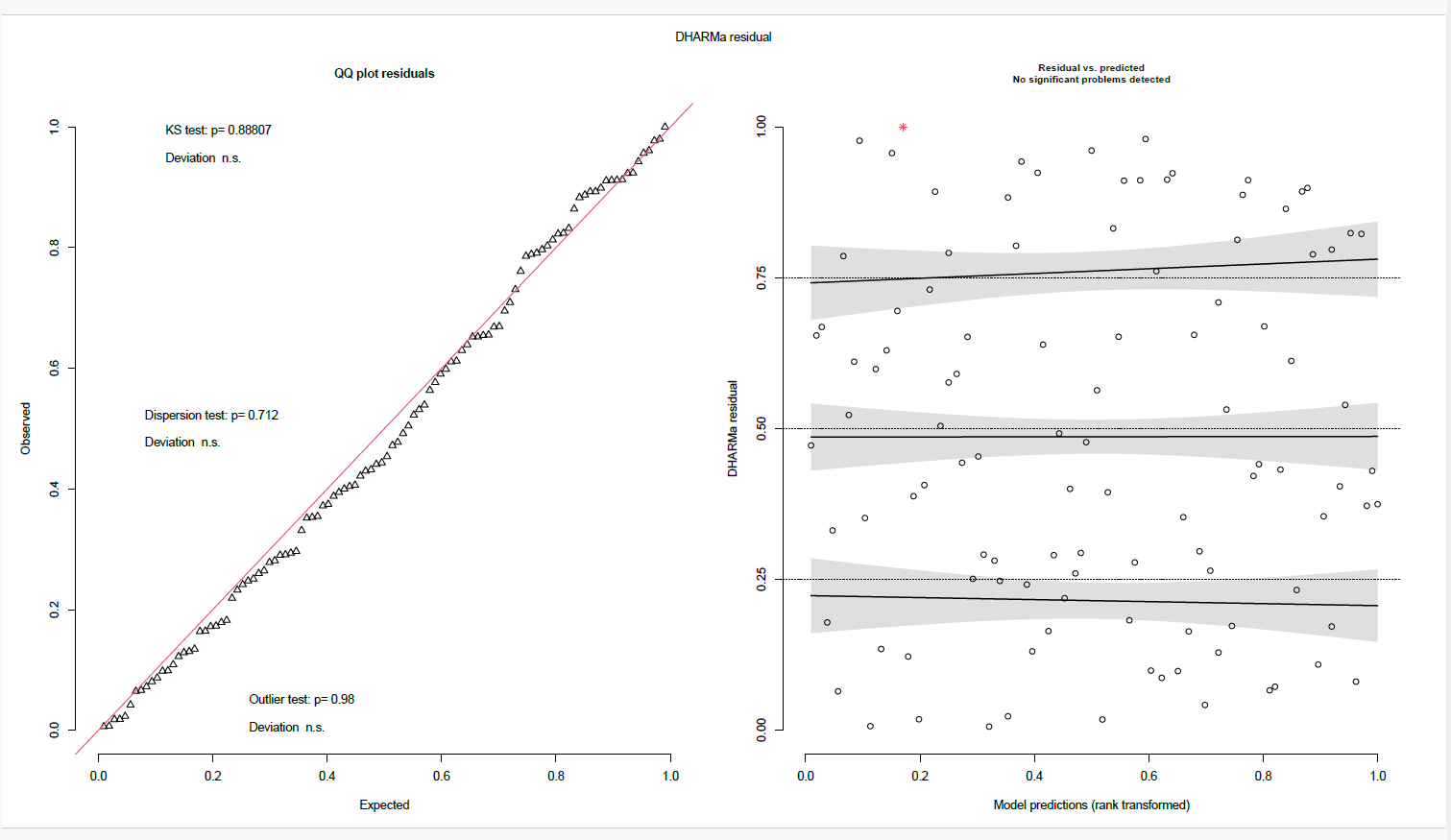


1. **Global score** model residuals using the “robustlmm” R package


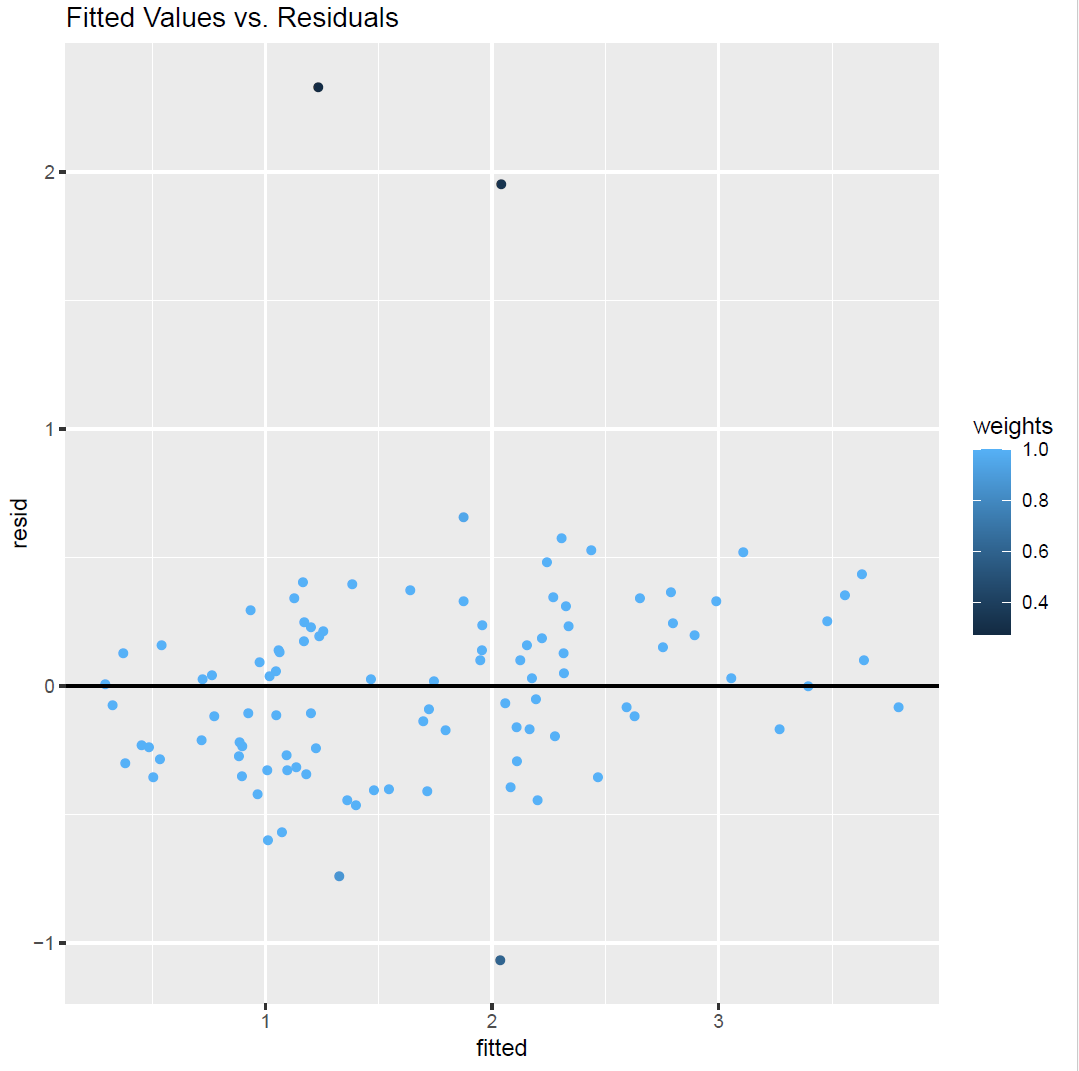


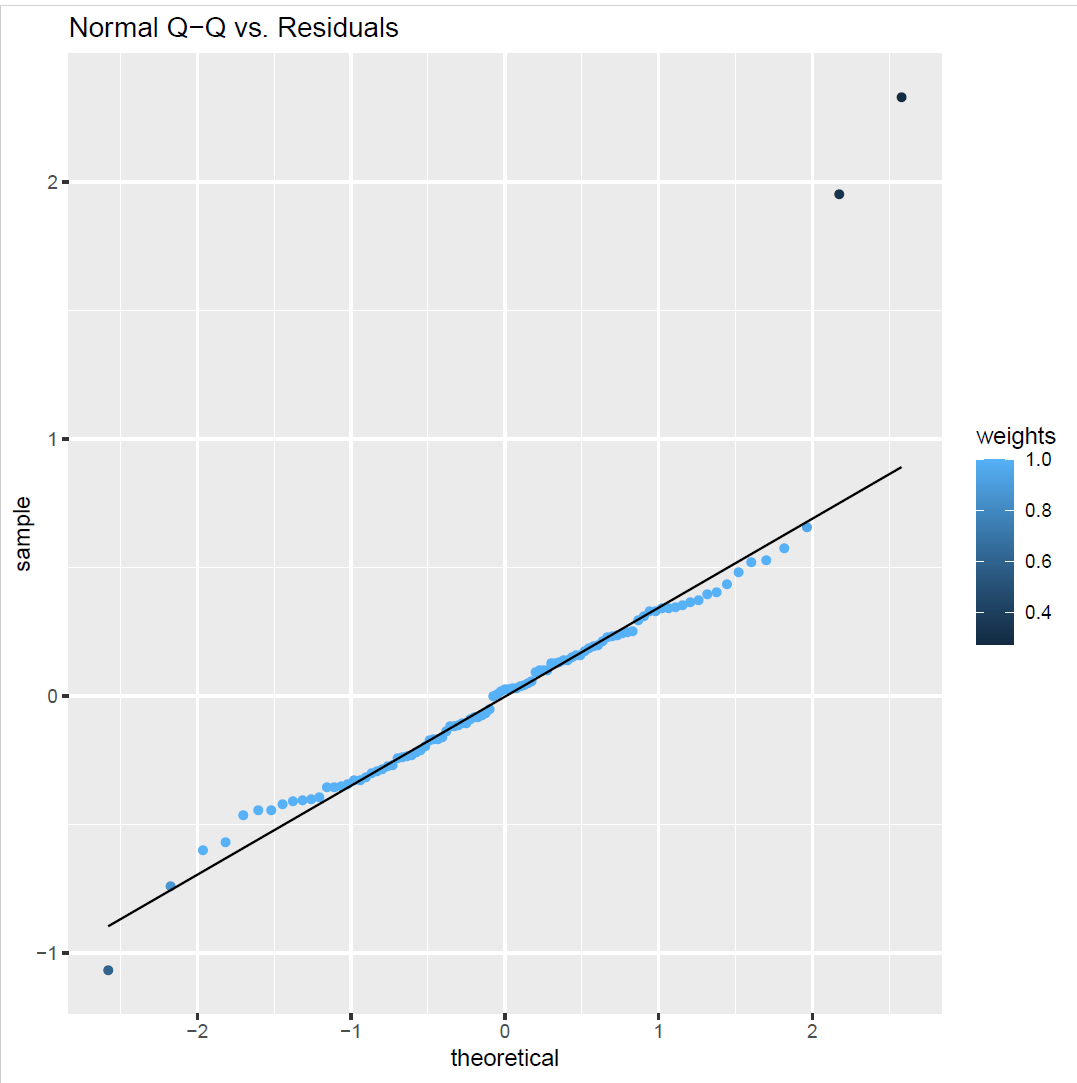


1. **BMI SDS** model residuals


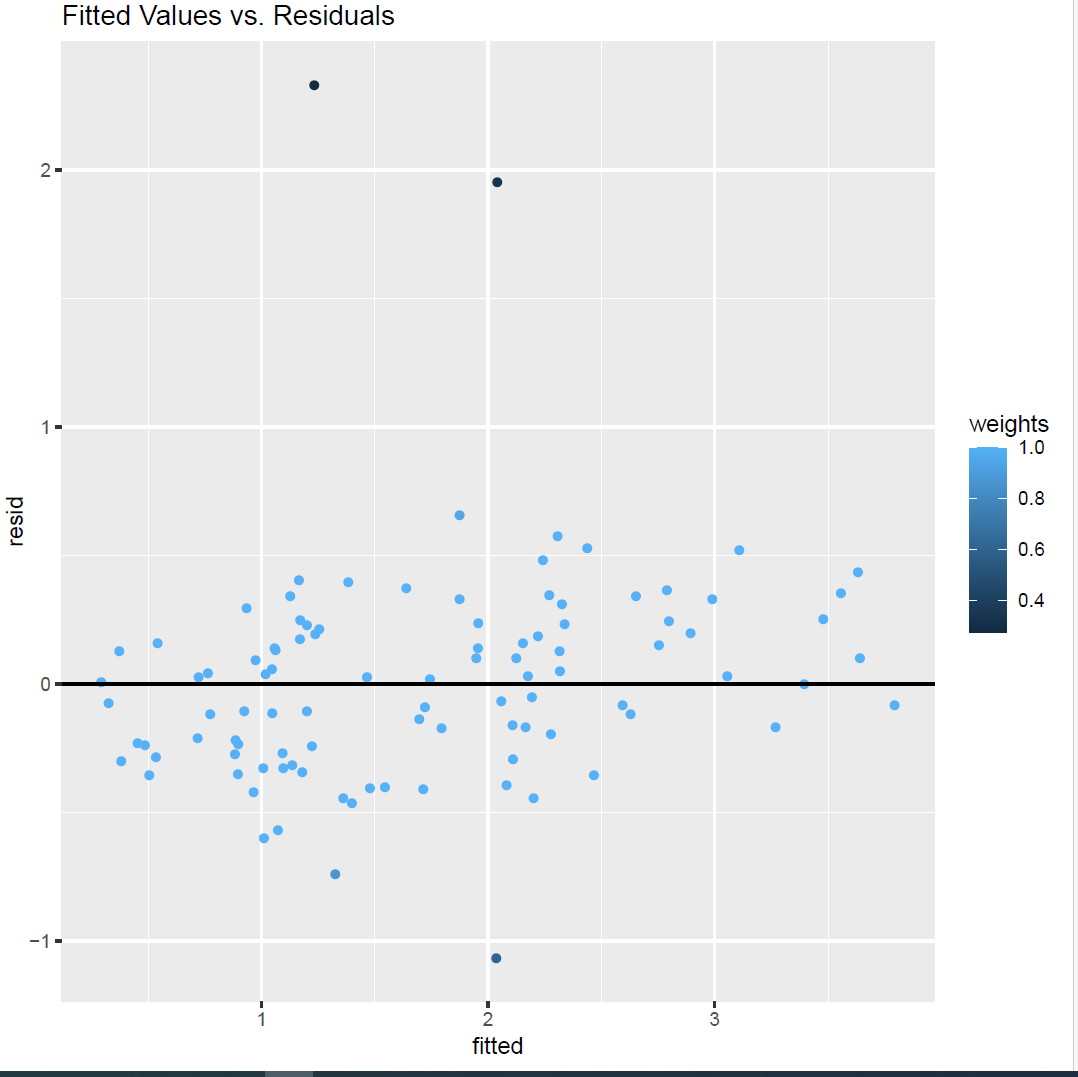


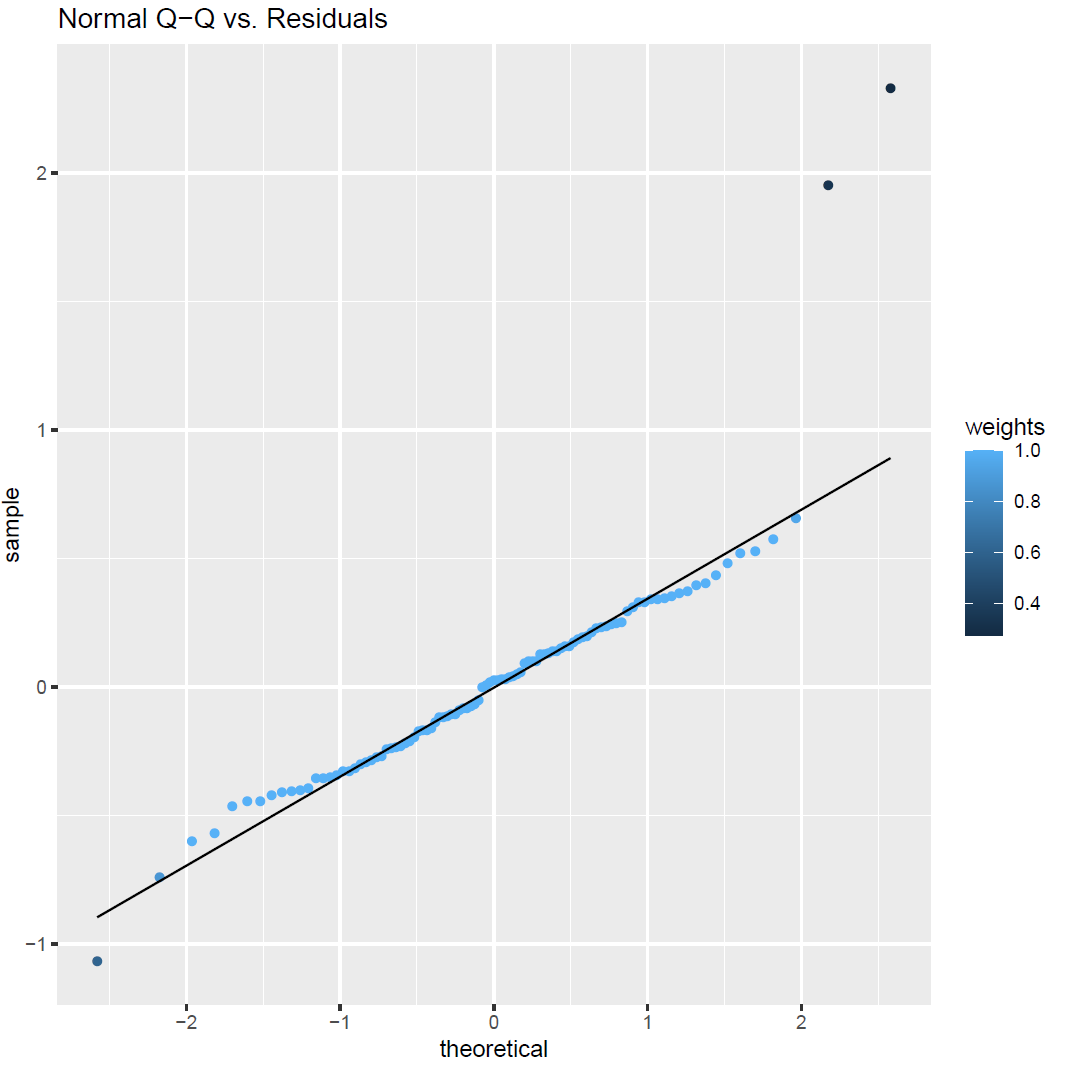


1. **Dietary restraint** model residuals


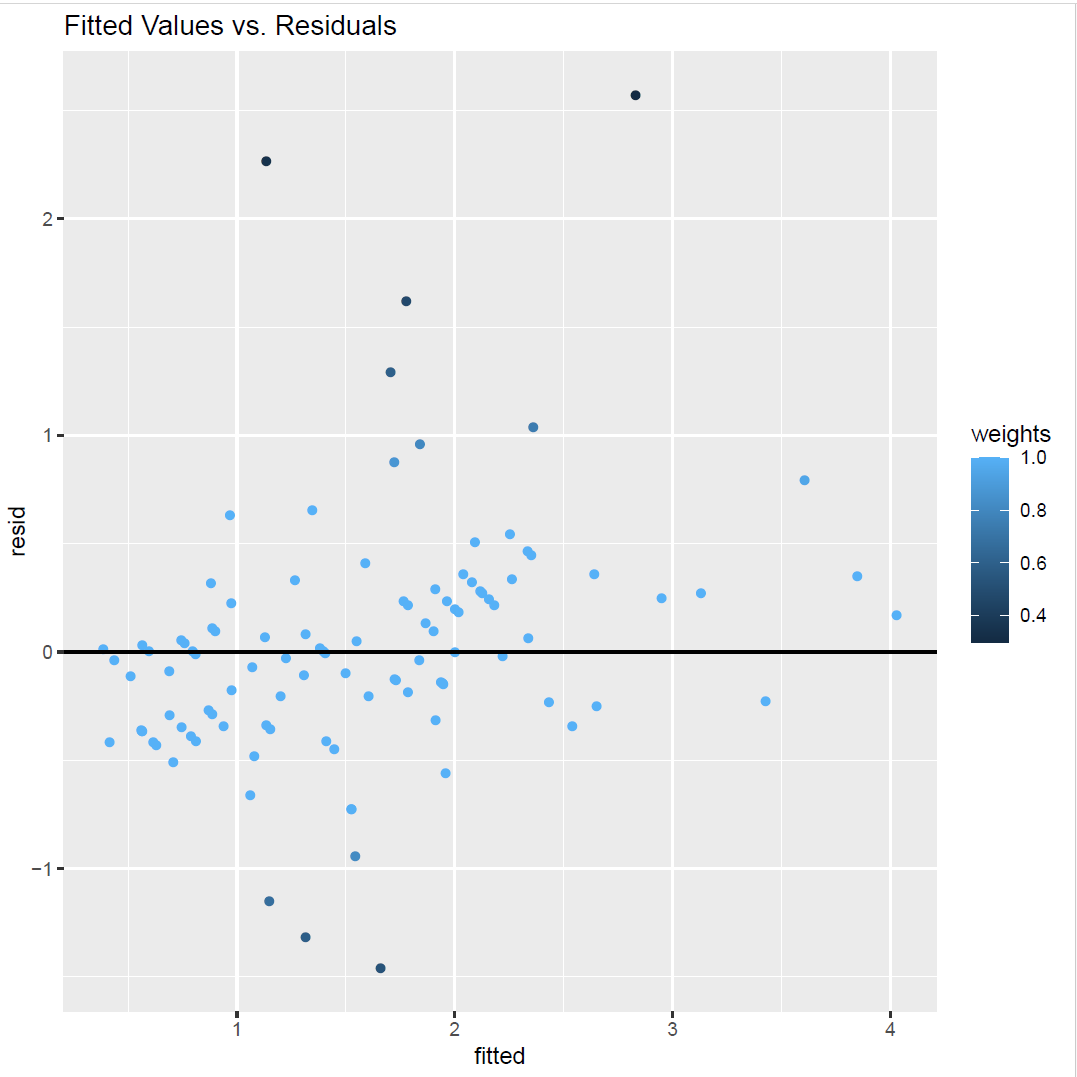


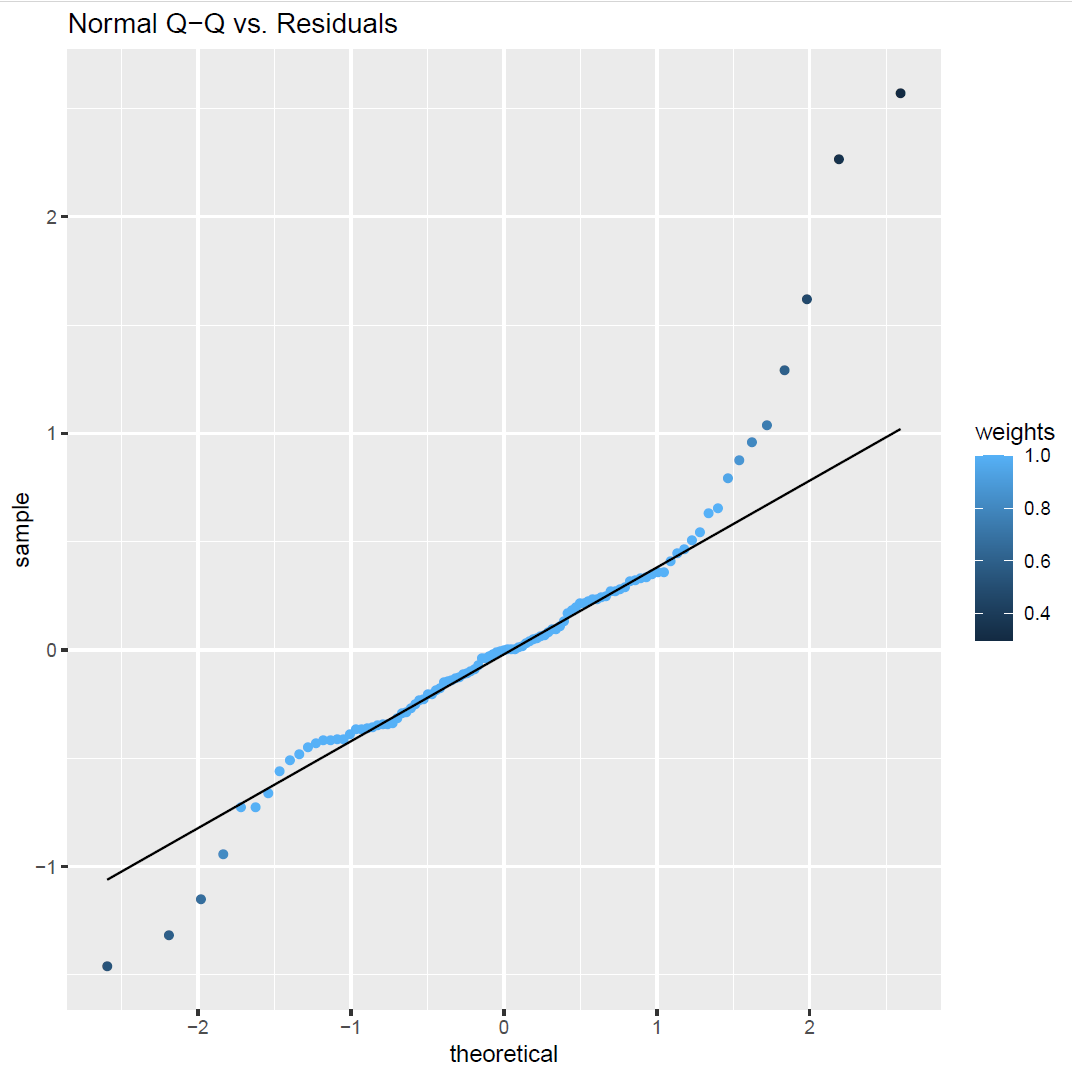


1. **Eating concern** model residuals


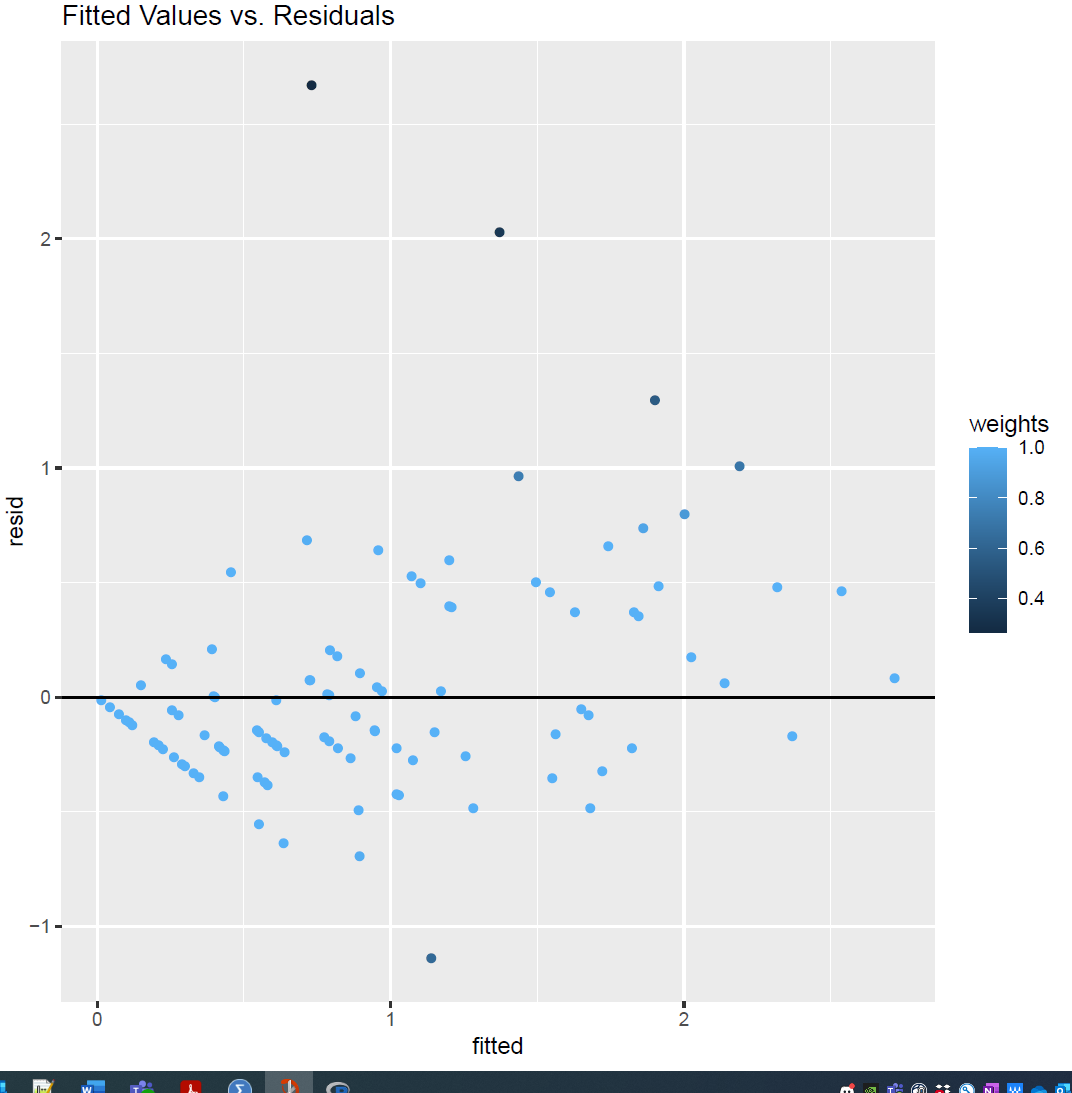


**
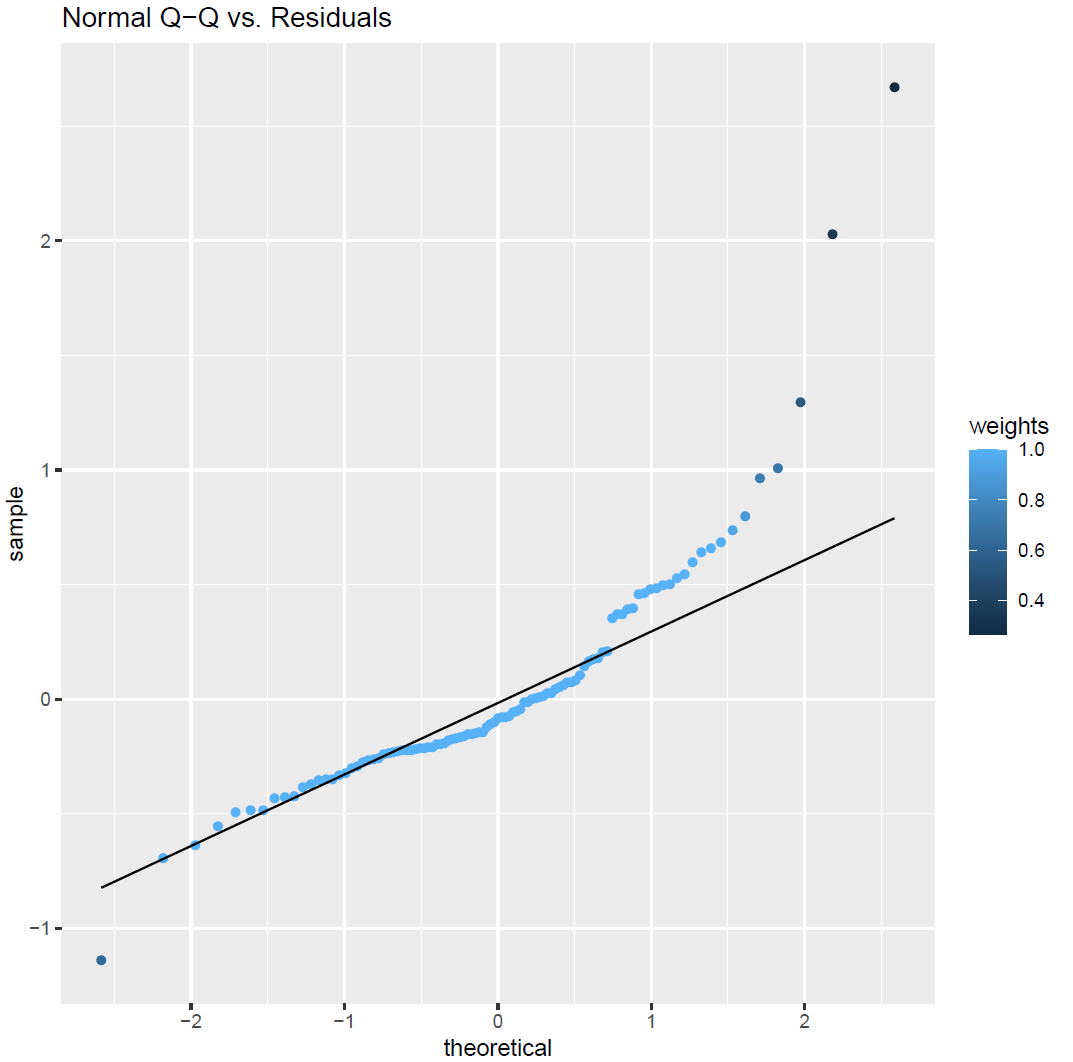
**

1. **Shape concern** model residuals

**
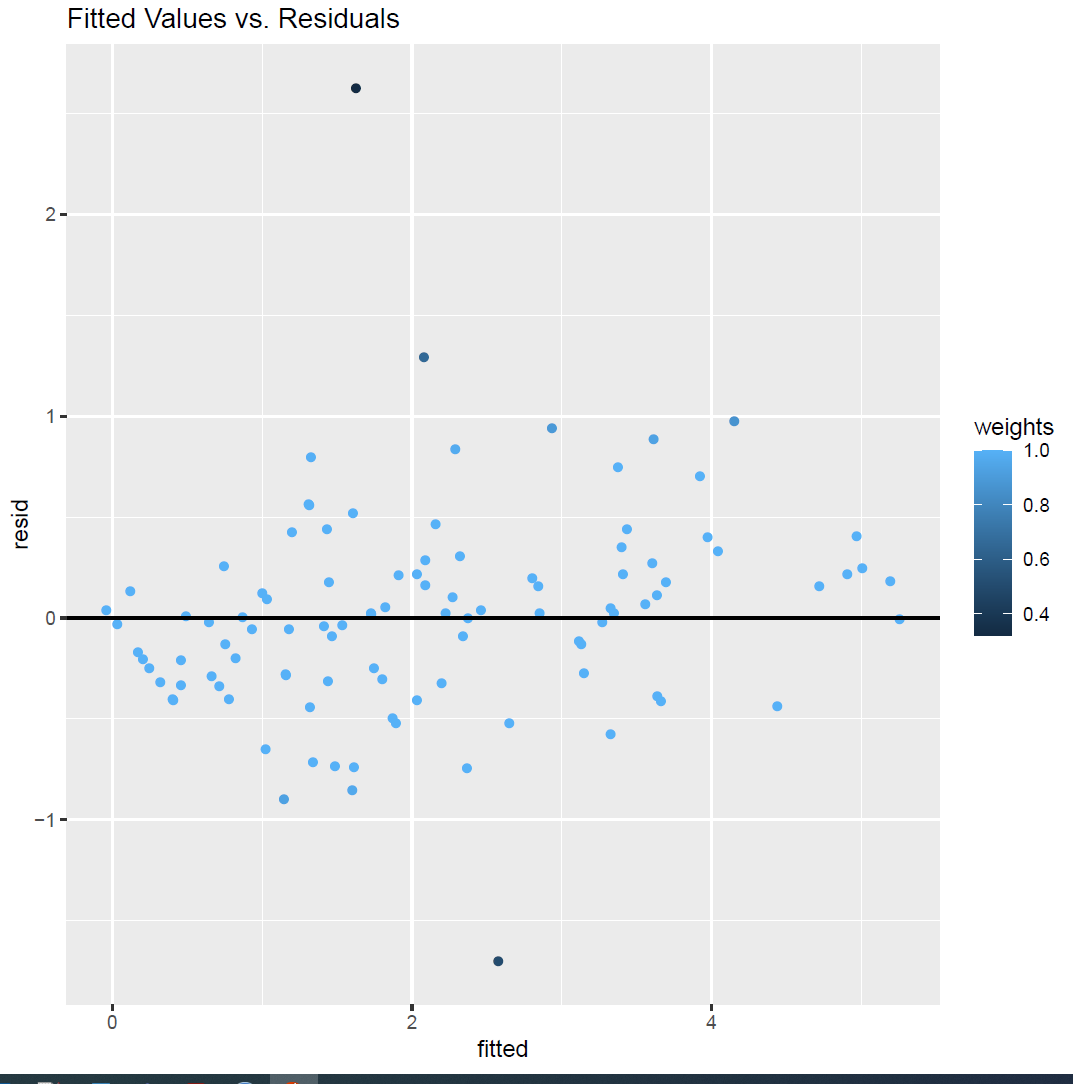
**

**
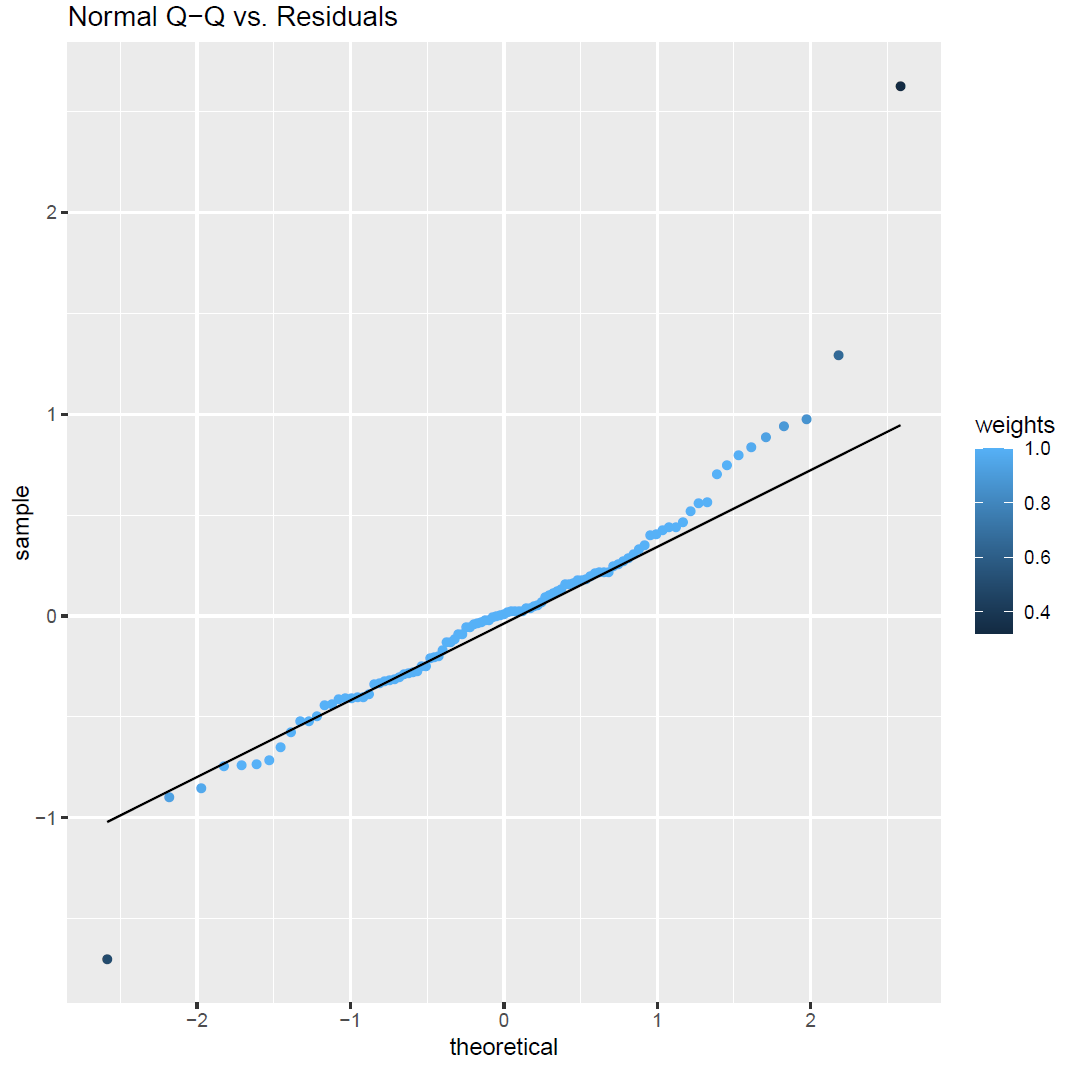
**

1. **Weight concern** model residuals


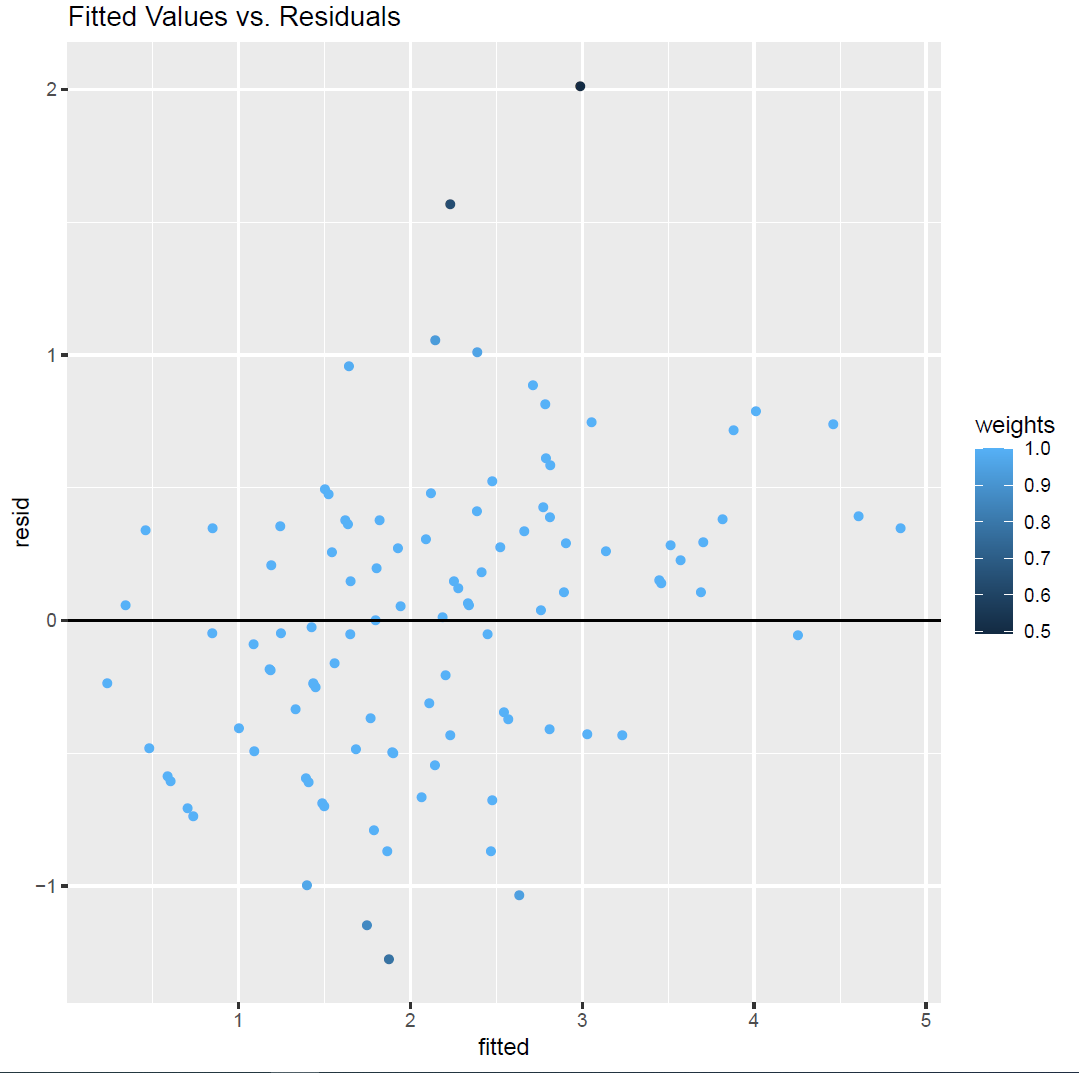


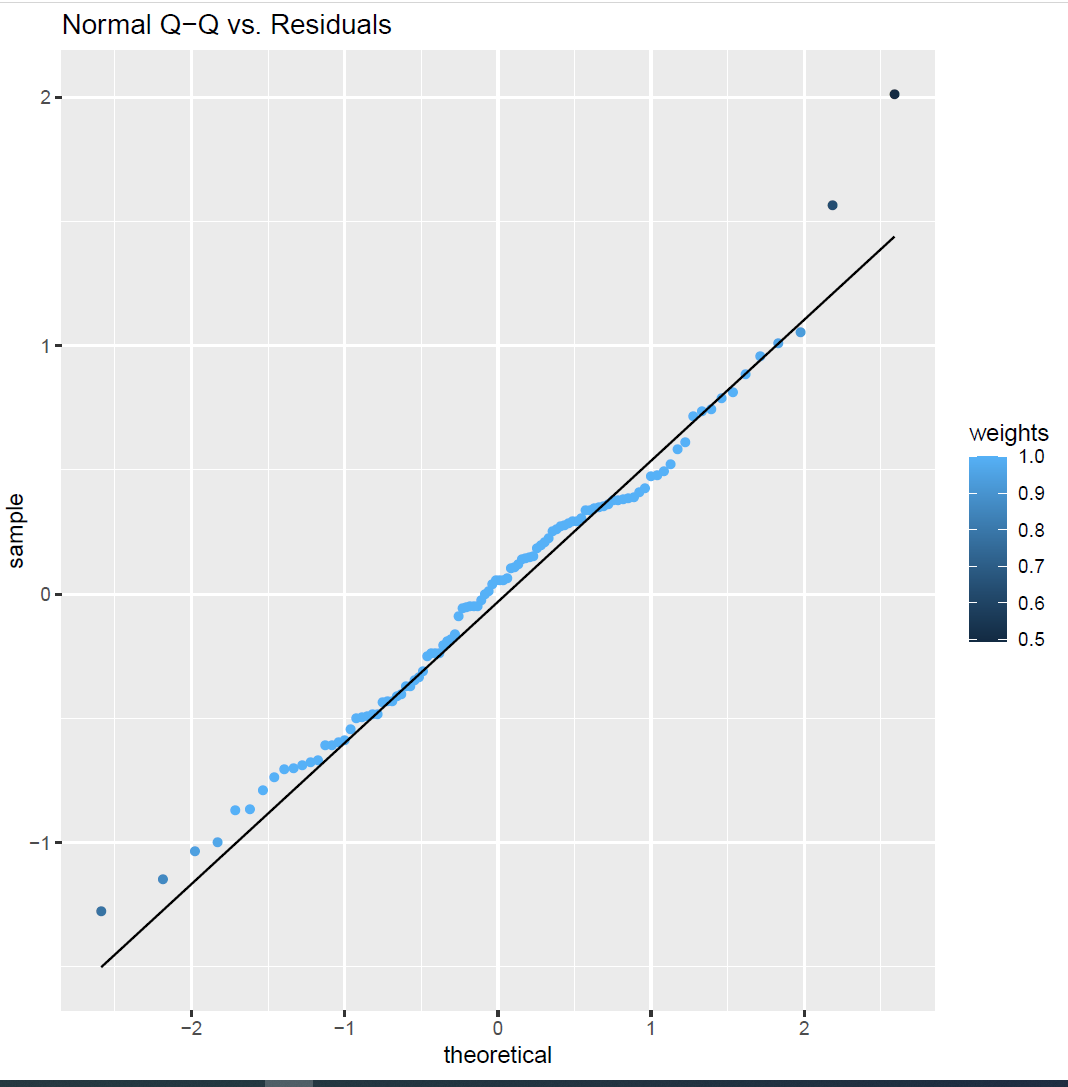

Supplement: Supplementary file 1 — Supplementary Material 1. [file 40519_2026_1839_MOESM1_ESM.docx]
