# Supplementary material for: Youth with severe obesity do not demonstrate increased eating disorder symptoms following family-based behavioral obesity treatment
Source: Eat Weight Disord. 2026 Mar 14;31(1):38. doi: 10.1007/s40519-026-01839-3 (PMC13099665; doi:10.1007/s40519-026-01839-3)

# Supplementary Material Appendix B

Y-EDE-Q Item Distributions by Subscale

## Figure S1. Restraint Subscale Items (Items 1,2,3,4 and 11)

Distribution of scores at T1 (baseline) and T2 (end of treatment).


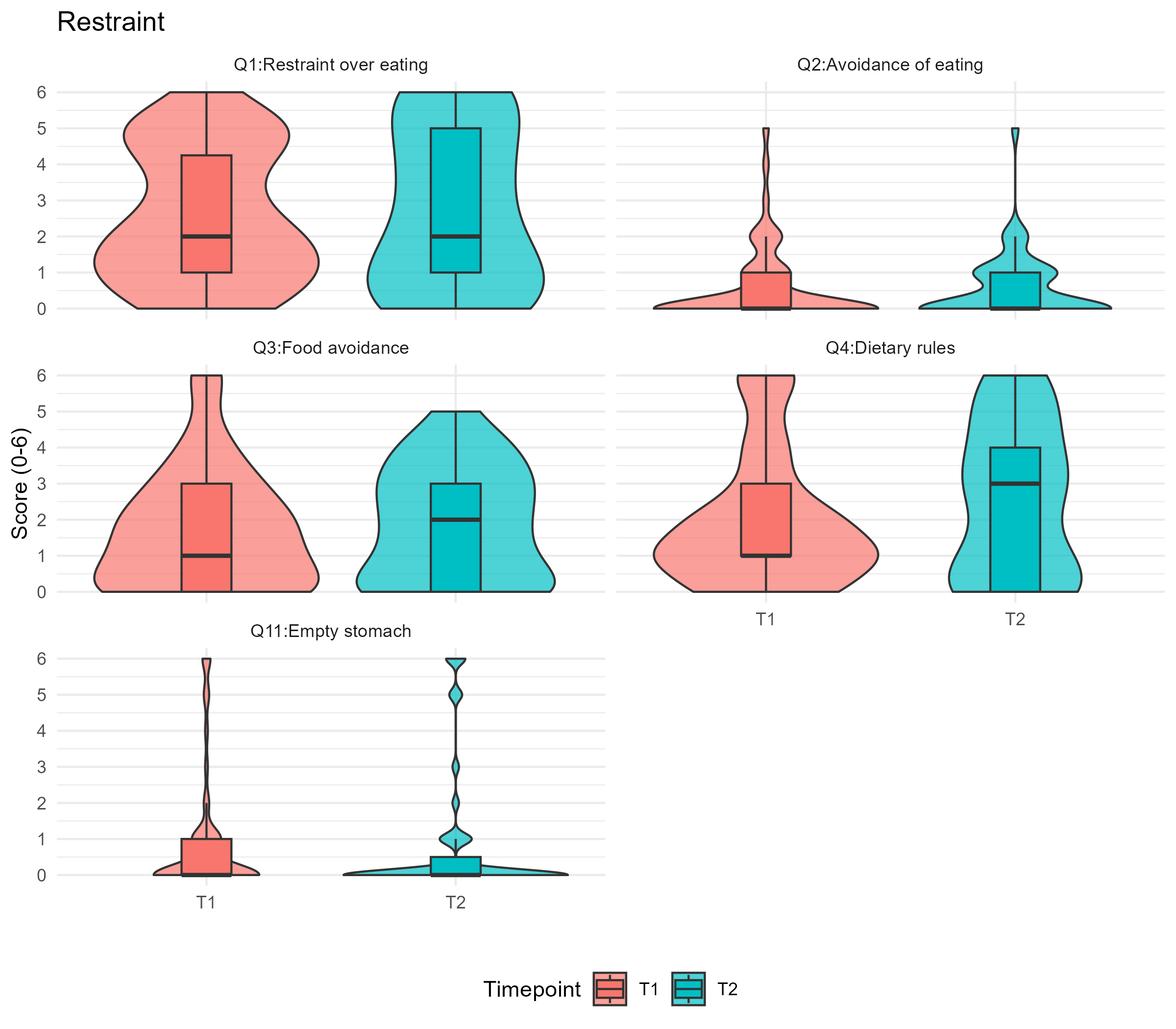


## Figure S2. Eating Concern Subscale Items (Items 5,6,9,16 and 36)

Distribution of scores at T1 (baseline) and T2 (end of treatment).


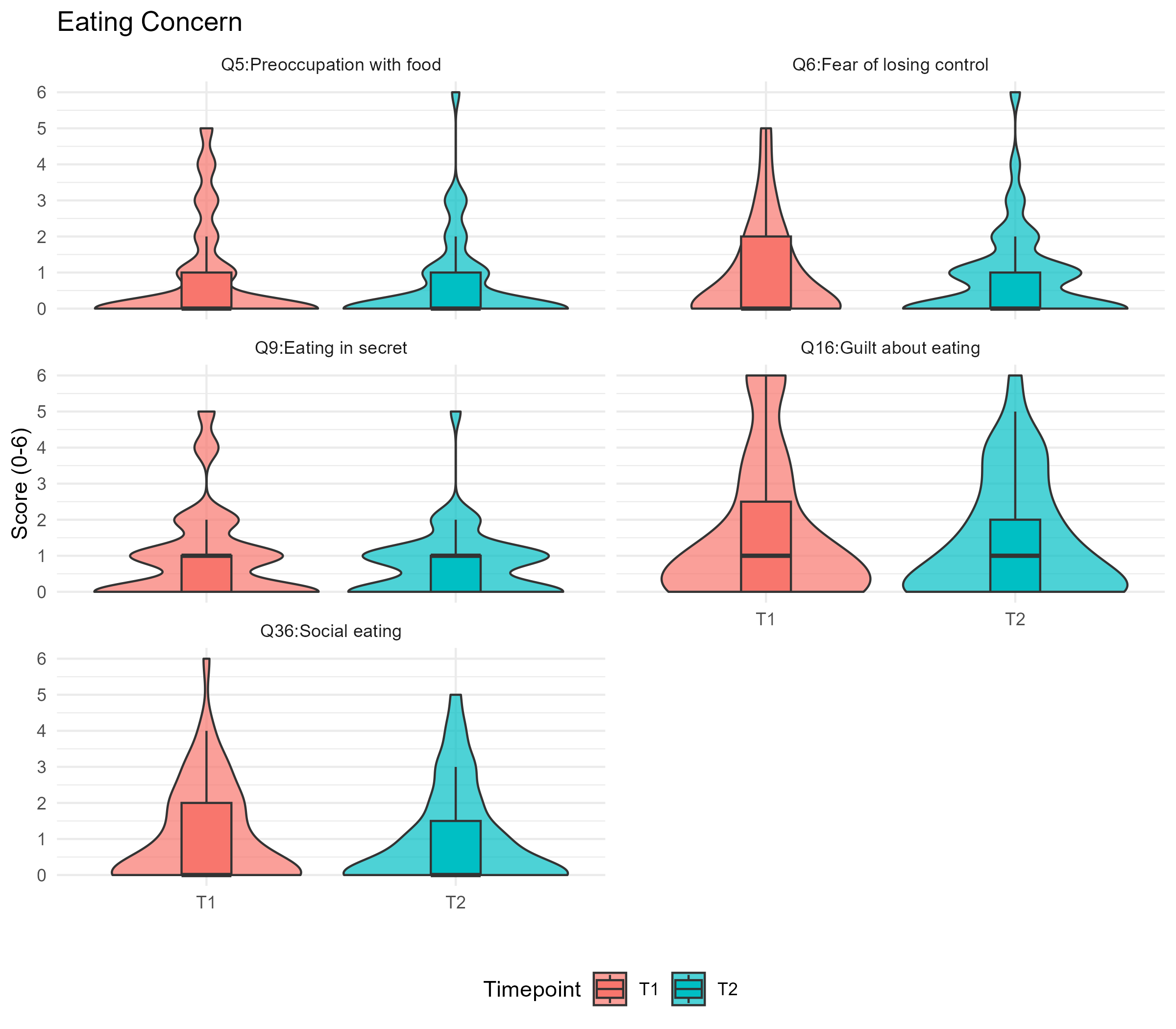


## Figure S3. Weight Concern Subscale Items (Items 12,15,30,32 and 33)

Distribution of scores at T1 (baseline) and T2 (end of treatment).


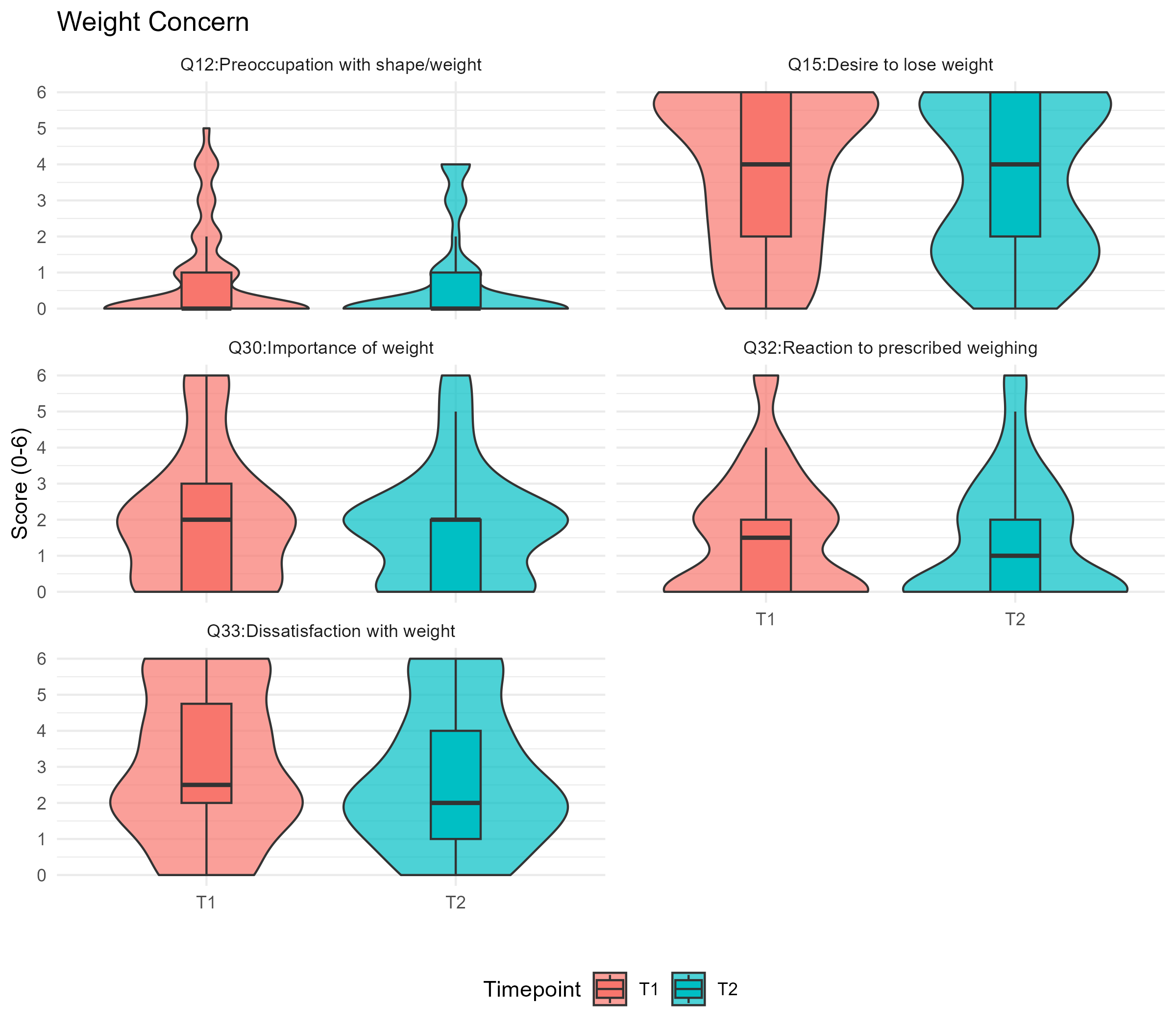


## Figure S4. Shape Concern Subscale Items (Item 10,12,13,14,31,34,37 and 38)

Distribution of scores at T1 (baseline) and T2 (end of treatment).


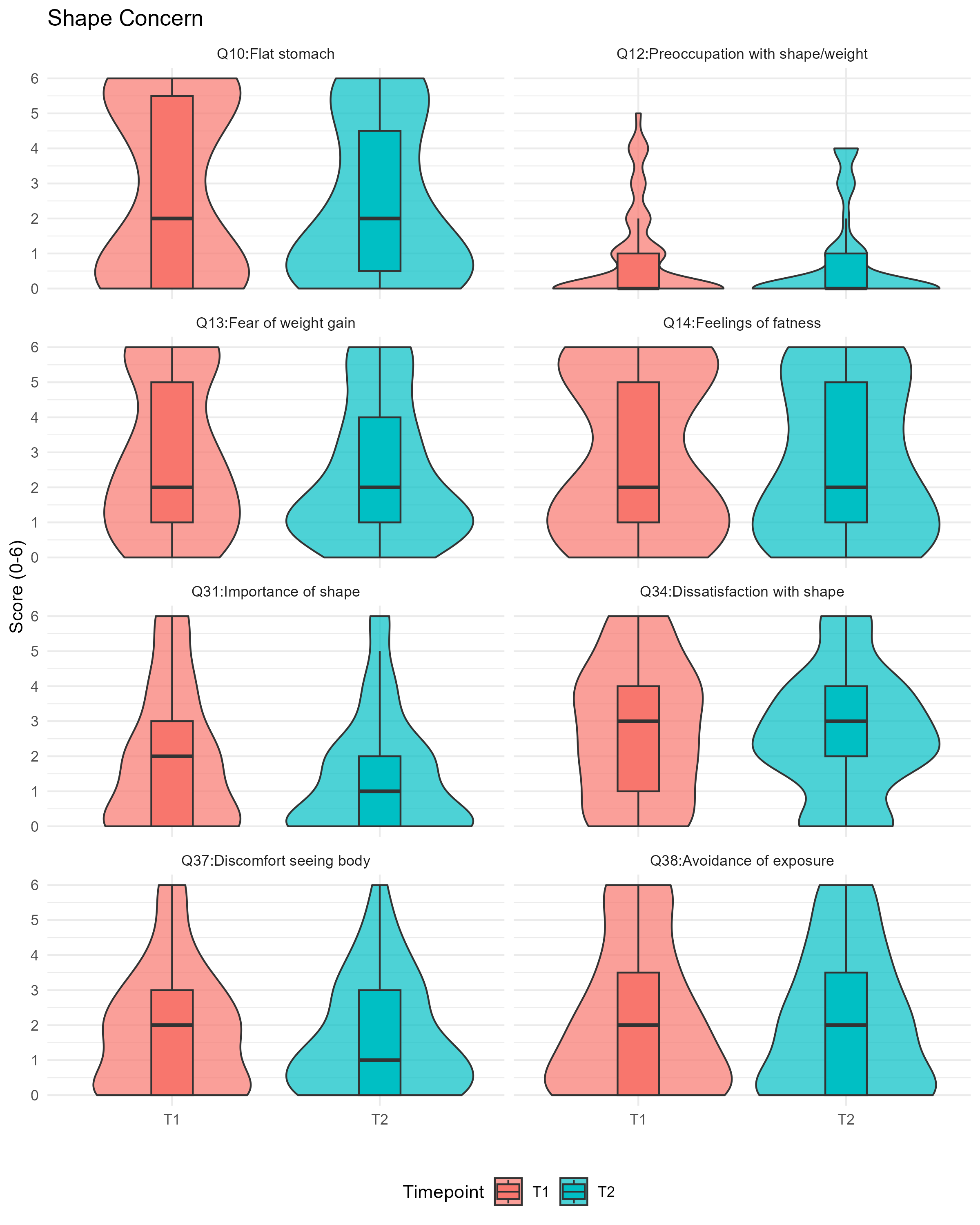

Supplement: Supplementary file 2 — Supplementary Material 2. [file 40519_2026_1839_MOESM2_ESM.docx]
